# Virtual Reality Rehabilitation for a Post‐ICU Hospitalized Patient With a Traumatic Brain Injury: A Single‐Patient Case Report

**DOI:** 10.1002/brb3.71686

**Published:** 2026-08-17

**Authors:** Jaylyn Aragon, Savanna Monson, Nathan Oh, Javier Romero, Graal Diaz, Bahareh Abbasi, Kristen Faye Linton

**Affiliations:** ^1^ Health Science Program California State University Channel Islands >Camarillo California USA; ^2^ Trauma Department Ventura County Medical Center Ventura California USA; ^3^ Mechatronics Program California State University Channel Islands >Camarillo California USA

**Keywords:** acute rehabilitation, traumatic brain injury, virtual reality

## Abstract

**Purpose:**

Visual impairment is common in individuals with traumatic brain injury (TBI). Virtual reality (VR) technology offers potential for acute rehabilitation by improving engagement and providing real‐time feedback. This single‐patient case report explores the feasibility and preliminary effects of VR for eye rehabilitation in a hospitalized, post‐ICU acute TBI patient.

**Method:**

A mid‐30s male with TBI participated in six sessions of the VR scenario “Beach Stroll.” Each session lasted up to six minutes and was conducted at the patient's bedside. The VR setup included an HTC Vive Pro Eye headset, which provided real‐time feedback on eye fixation. Data on blood pressure, heart rate, pain, and anxiety were collected before and after each session. Patient feedback was also documented.

**Finding:**

Patient reported that the VR sessions reduced anxiety and increased comfort. The patient described the experience as “the coolest thing I've done here [in the hospital],” with “really soothing” sounds and “low stimulation.” Objective measures showed stable or decreased blood pressure and heart rate and decreased pain and anxiety across sessions.

**Conclusion:**

The positive trends observed in this novel single‐patient case report suggest the feasibility and acceptability of brief VR eye‐tracking rehabilitation in the acute post‐ICU hospital setting for a patient with TBI.

## Graphical abstract

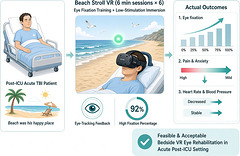

## 1 Virtual Reality Rehabilitation for a Post‐ICU, Hospitalized Patient With a Traumatic Brain Injury: A Case Report

Visual impairment in individuals with traumatic brain injury (TBI) is a significant concern present in 32%–90% of people with TBI (Samadani et al. 2015; Fox et al. 2019). TBI, including its milder form, mild traumatic brain injury (mTBI), is a major cause of disability and morbidity, particularly affecting vision. The prevalence of visual symptoms and dysfunctions in TBI patients is high, with a variety of visual problems reported, such as defects in primary vision (visual acuity, visual fields), eye movement dysfunctions (fixation‐ quickly and accurately looking, vergence, saccadic‐ moving eyes from point to point, and smooth pursuit movements‐ smoothly following an object), and more complex aspects of vision (visual perception, motion vision, and visuo‐spatial function) (Capó‐Aponte et al. 2017; Armstrong 2018; Samadani et al. 2015). Visual dysfunction and impairment hinder patients’ ability to be successful in physical and cognitive rehabilitation (Fox et al. 2019).

Eye fixation, the ability to focus on objects, in TBI patients is a commonly reported challenge. Eye movement dysfunction is reported in about 90% of acute patients suffering from concussion or blast injury (Samadani et al. 2015). Long‐term vision dysfunction is experienced by 32%–79% of people with TBI (Fox et al. 2019). Problems with vergence eye movements, which are binocular movements tracking objects in depth, are particularly prevalent. Convergence insufficiency (CI), where the eyes may drift outward during close work and struggle to work together when focusing on close objects (eye fixation) leading to eye strain, blurred vision, and diplopia (double vision), is especially prevalent in TBI patients. This condition can lead to symptoms like eye strain, blurred vision, and diplopia (double vision) (Armstrong 2018; Fox et al. 2019). This condition is linked to slower reading speeds, compromised attention, impaired performance at work, and having conversations with others (Armstrong 2018; Fox et al. 2019; Samadani et al. 2015). A systematic review of oculomotor deficit interventions for people with brain injury found “few studies overall,” with only six of nine total studies having addressed fixation (Watabe et al. 2019). Another systematic review of 22 studies of visual research on mild TBI, visual fixation research was focused on the least (*n* = 3; Stuart et al. 2020). One recent study conducted a randomized controlled pilot of an eye‐tracking VR rehabilitation among people with brain injury in the outpatient setting (Linton et al. 2024).

### 1.1 Eye Tracking and Virtual Reality Technology

New technology in virtual reality (VR) headsets (such as the HTC Vive Pro Eye 2019) with pupil tracking capabilities has provided an opportunity to develop portable, affordable rehabilitation systems (Aulisio 2020). Three main strengths to using VR for eye tracking are that it has a wide visual field and high temporal precision, it has potential for diverse applications, and it has innovative testing methodologies. The HTC Vive Pro Eye virtual reality headset can test across a wide visual field of the head‐mounted display's screen, demonstrating good temporal precision, crucial for applications like VR perimetry and as an online assistance tool for visual loss (Sipatchin et al. 2021). The HTC Vive Pro Eye accuracy and precision were highest for central gaze (Schuetz and Fiehler 2022). VR eye‐tracking technology has been used in various fields, including sports, healthcare, and rehabilitation, highlighting its versatility and potential for patient‐friendly clinical applications (Sipatchin et al. 2021). Novel testing methods and setups, such as the use of a Raspberry Pi system for temporal precision testing, have been developed, showcasing the adaptability and innovation in VR eye‐tracking research (Sipatchin et al. 2021).

The four main weaknesses in using VR for eye tracking are spatial accuracy concerns, influence of head movement and facial configuration, challenges with reflections and light condition, and calibration concerns. The spatial accuracy of the HTC Vive Pro Eye's eye‐tracking system, especially at the peripheral vision areas, has been found to be less accurate than the manufacturer's claims, with significant inaccuracy observed at targets positioned on the upper horizontal line (Sipatchin et al. 2021). The accuracy and precision of eye‐tracking data are affected by head movement and subjective facial configurations, such as the distance of the VR headset from the eyes, which can shrink the visual field and make fixation in certain areas more challenging (Sipatchin et al. 2021). Eye‐tracking data quality can be compromised by reflections from the surrounding environment, the use of corrective lenses, and the position of the infrared camera inside the VR headset (Sipatchin et al. 2021). Current calibration methods might not be sufficient in scenarios involving head movement, suggesting a need for novel calibration approaches, especially in dynamic VR environments (Sipatchin et al. 2021).

In conclusion, VR eye‐tracking using the HTC Vive headset offers a promising tool with its wide visual field, high temporal precision, and potential for diverse applications. However, challenges in spatial accuracy, especially in peripheral vision and under head movement, along with issues like reflections and the need for better calibration methods, highlight areas for future improvement and research.

### 1.2 Virtual Reality and Rehabilitation

Use of VR for rehabilitation can provide an enhanced rehabilitation experience, increased engagement and motivation, real‐time feedback, potential for home‐based rehabilitation, and customization. VR provides an immersive, interactive environment that can be tailored to specific rehabilitation needs (Jiménez‐Rodríguez et al. 2023). Due to its novelty and gamification, the engaging VR can enhance patient motivation and participation in rehabilitation exercises. Eye‐tracking technology in VR headsets, like the HTC Vive, offers feedback in the moment, which can improve the effectiveness of rehabilitation exercises (Park et al. 2019). VR technology could allow for more accessible home‐based rehabilitation options, reducing the need for frequent clinic visits and addressing disparities related to rehabilitation (Jiménez‐Rodríguez et al. 2023). VR systems can offer a wide range of exercises and activities, which can be customized to the individual needs of the patient.

VR for rehabilitation is limited in technology, cost, lack of standardized protocols, knowledge on long‐term efficacy, physical discomfort and safety, and limited applicability for certain patient groups. The high cost of VR equipment and technical limitations, such as the accuracy and precision of eye‐tracking, can be barriers to widespread use at this time; however, as the technology advances, costs are expected to reduce over time (Jiménez‐Rodríguez et al. 2023; Schuetz and Fiehler 2022). There is a lack of standardized protocols for VR rehabilitation, leading to variability in treatment approaches and outcomes (Raphanel et al. 2018). There is a need for more research on the long‐term efficacy of VR in eye rehabilitation, particularly in diverse patient populations (Schuetz and Fiehler 2022). Some patients may experience physical discomfort, such as dizziness or nausea, when using VR headsets. Safety concerns during unsupervised use are also notable. The effectiveness of VR may be limited for patients with specific conditions or those with severe visual impairments (Raphanel et al. 2018). In conclusion, while VR, particularly with devices like the HTC Vive, shows promise in eye rehabilitation, challenges such as cost, standardization, and research on long‐term outcomes need to be addressed. Future developments in VR technology and more rigorous clinical trials could further establish its role in rehabilitation therapy.

### 1.3 Virtual Reality and TBI Vision Rehabilitation

Strengths of virtual reality in vision rehabilitation among people with TBI include the length of time and frequency of the intervention. Nine studies have consistently found that vision dysfunction can be improved by 6–8 weeks of twice‐weekly 20‐60‐minute VR sessions (Watabe et al. 2019). Weaknesses in the rigor of studies on virtual reality on vision rehabilitation among people with TBI include a limited number of studies, limited experimental studies, small sample sizes, lack of diversity in the samples, lack of assessment of enduring effects, and no research conducted in the acute, clinical setting. Only ten studies have been conducted on the effectiveness of virtual reality on vision rehabilitation among people with TBI (Watabe et al. 2019; Linton et al. 2024). Four studies used experimental designs, while six were case studies (Watabe et al. 2019; Linton et al. 2024). Sample sizes ranged from 2–40. Demographics of studies beyond their brain injury information are limited. Minimal studies mention language translation of materials or racial/ethnic demographics of participants besides Linton et al. (2024). All studies on VR and vision rehabilitation except one (Linton et al.) were conducted in a laboratory setting rather than an acute hospital or community‐based setting. Research in acute medical settings with larger, diverse samples is needed to test the efficacy of VR on vision rehabilitation among people with TBI.

Marklund and colleagues’ (2019) summarized common rehabilitation offerings to people with TBI in the acute and subacute stages of recovery, which commonly only included physical and occupational therapy. Virtual reality could expand rehabilitation options to include vision rehabilitation available in the hospital setting for patients with TBI. Common acute and subacute rehabilitation includes therapies that increase neuroplasticity (Marklund et al. 2019). Since 70% of the brain is used in visual processing, participants focusing their eyes on an object could improve neuroplasticity necessary for optimal recovery (Padula 2017; Suter 2017). From a neurobiological perspective, VR‐based visual stimulation may facilitate recovery after TBI through several interconnected mechanisms. The immersive and multisensory nature of VR can promote neuroplasticity by providing enriched environments that encourage repeated activation of visual and attentional networks (Zanier et al. 2018; Aida et al. 2018). Focused eye movements on a salient target, as designed in the Beach Stroll scenario, may engage frontoparietal attentional networks and support sensorimotor integration, potentially aiding recalibration of visuomotor pathways disrupted by injury (Adamovich et al. 2009; Musick and Yalcin 2025). Additionally, the combination of visual engagement with a relaxing yet alerting immersive environment may help modulate autonomic regulation through connections between visual processing areas and limbic/emotional centers. This is particularly relevant in the acute post‐ICU phase, when elevated anxiety is common and can further impair attention and visual performance (Delmonico et al. 2022).

Previous studies of virtual reality rehabilitation for people with traumatic brain injuries have focused on the rehabilitation but have not combined the data that shows that virtual reality can be relaxing and improve mental health (Watabe et al. 2019). In acknowledging that, especially for acute, hospitalized patients, people with TBI may have increased anxiety (Delmonico et al. 2022). Delmonico et al. (2022) found that over 20% of people with TBIs also have co‐occurring mental health issues in the first‐year post‐injury.

### 1.4 Justification for This Case Study

Previous research on VR eye tracking among people with TBI has primarily used laboratory settings (Watabe et al. 2019). This single‐case study was conducted in collaboration with a local trauma hospital and a local brain injury support nonprofit organization. The patient was identified and recruited at a local trauma hospital where he received the VR intervention. Previous research has found that 20 to 60‐min sessions of VR eye‐tracking scenarios improved outcomes (Watabe et al. 2019). This study tested the effects of shorter 6‐minute sessions in the acute hospital setting inspired by focus groups with local patients with TBI and a familiar, relaxing setting for patients. This case study tested the effects of a bilingual (Spanish & English) virtual reality scenario our team developed, Beach Stroll/*Paseo por la playa*, on visual fixation among an acute, hospitalized patient with a TBI to determine its feasibility and rehabilitative effects at the hospital bedside.

## 2 Methods

### 2.1 Recruitment

The study received Institutional Review Board approval (# I05625) to be conducted by Ventura Country Medical Center and California State University Channel Islands. Potential participants were identified by a daily review Monday‐Friday of medical charts and consultation with physicians of eligible current patients admitted to a local trauma hospital, Ventura County Medical Center, by an administrator in quality improvement and data analytics. All eligible participants were presented with the option to participate in the study by an administrator in Quality Improvement and Data Analytics at Ventura County Medical Center. The informed consent was presented to eligible participants. Inclusion criteria included: 1. The patient that had a TBI and was hospitalized as a trauma patient at Ventura County Medical Center, 2. The patient was admitted to medical/surgical or step‐down ICU, 3. Patient had the capacity to consent, or a guardian who could sign an assent form, 4. The C‐spine was cleared by a neurosurgeon or trauma surgeon and had no limitation with moving the neck/spine, and 5. The patient was 12 years of age or older. Exclusion criteria were: 1. Patient was unable to consent, 2. Patient didn't have a TBI, or 3. Patient had neck or face injuries. Three patients were screened, one was ineligible due to still remaining in ICU, and a third was interested, but when the intervention was offered, the patient chose not to participate.

### 2.2 The Intervention‐Beach Stroll/*Paseo por la Playa*

The participant received the 6‐min Beach Stroll virtual reality scenario in the hospital setting. The participant was offered a beach stroll at the hospital bedside. A focus group with people with TBIs conducted to design the scenario found that people with TBI desired an outdoor, relaxing vibe, which inspired the creation of Beach Stroll (Linton et al. 2024). The Beach Stroll scenario was created by a collaboration between a clinician researcher with her MSW, a mechatronics professor, and a computer science undergraduate student using real film from a local southern California beach that is familiar to many of the local study participants. The participant heard waves crashing and seagulls chirping softly as they saw themselves (first‐person) slowly walking along the shore. Health science undergraduate students were virtual reality technicians who visited the patient and invited him to do beach stroll. The virtual reality technicians were supervised by a clinician researcher with her MSW.

**FIGURE 1:**
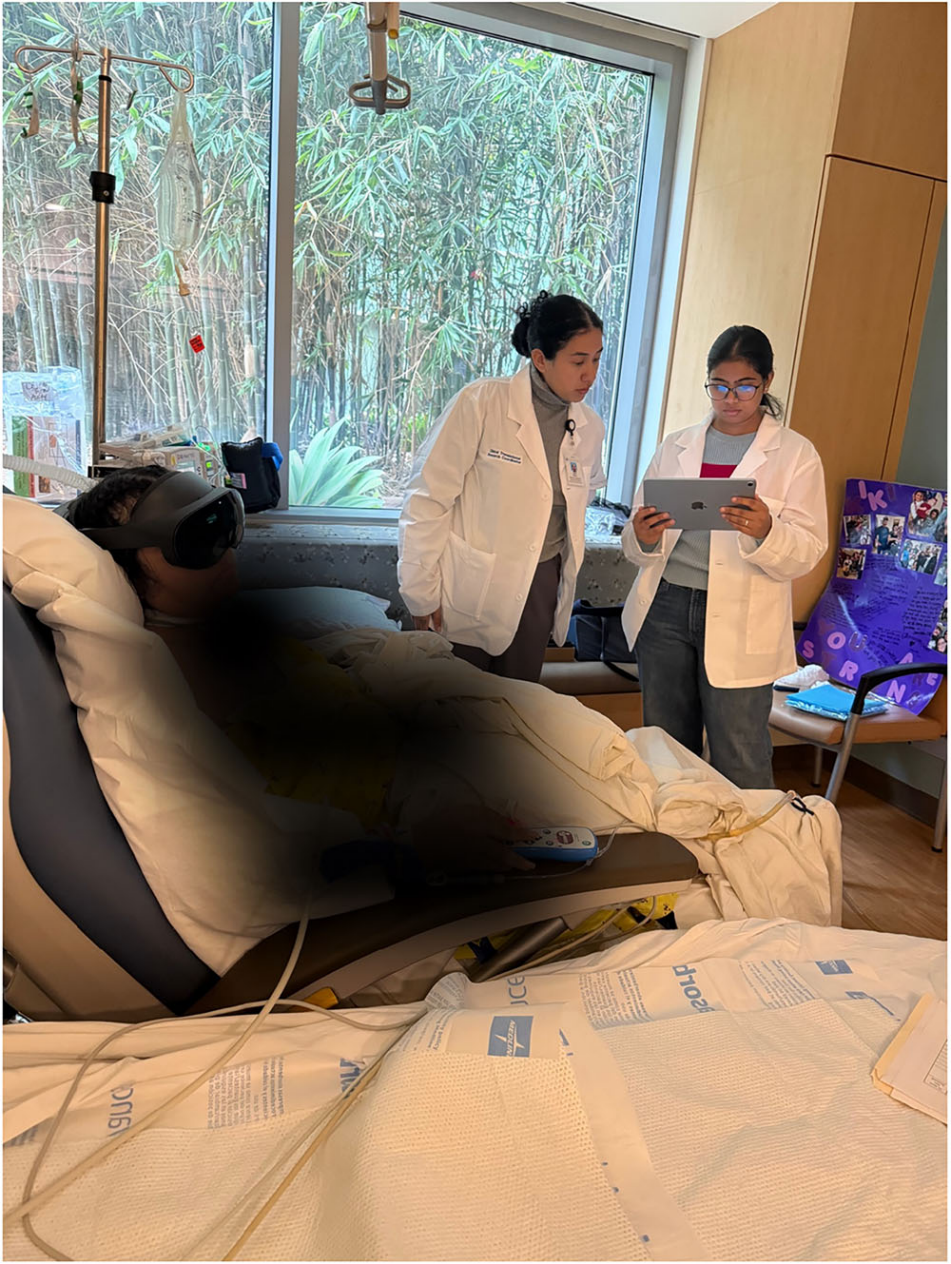
The Patient's Virtual Reality Headset Setup at Hospital Bedside. Alt text: Two research assistants holding an iPad while a participant is in a hospital bed with the headset over her face. Her body and the rest of her face are blurred.

**FIGURE 2:**
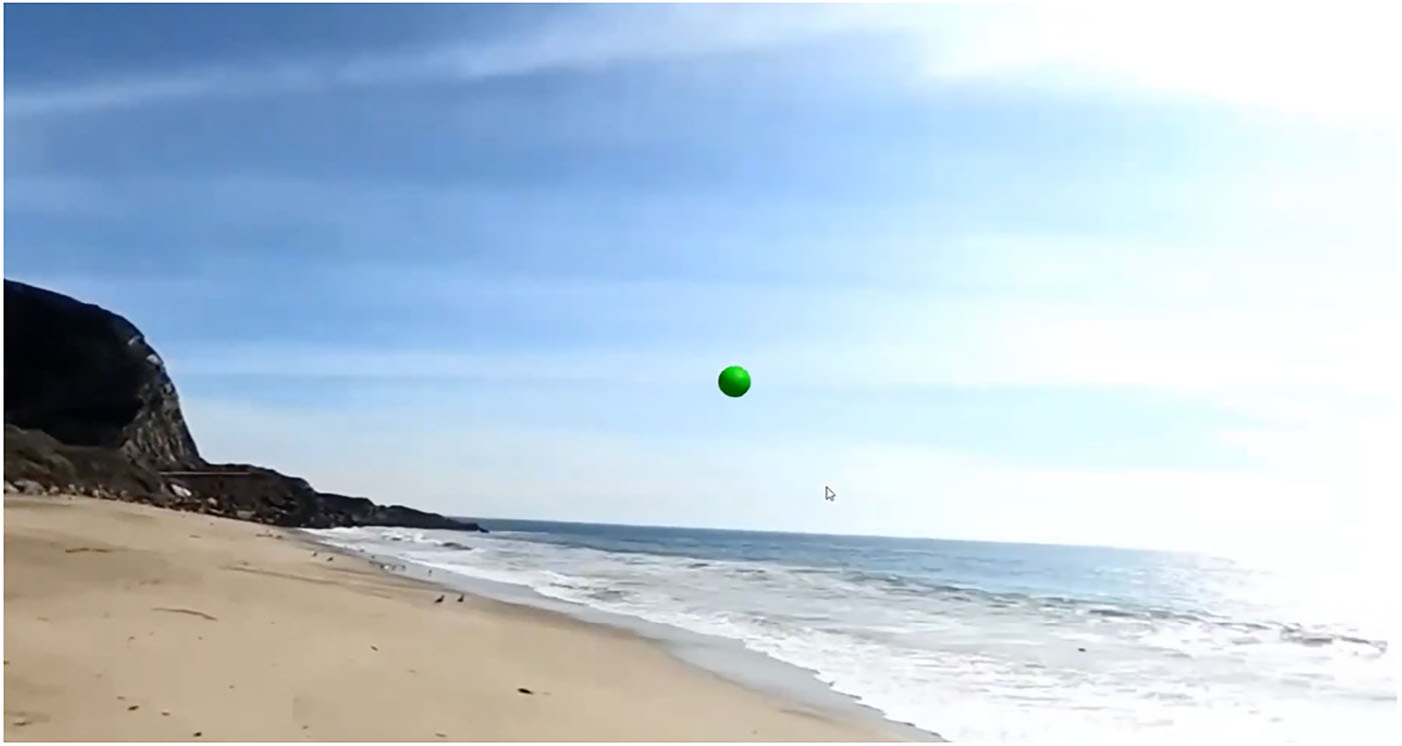
What the Patient Sees in the Beach Stroll Scenario. Alt text: The scenario includes the view of the ocean to the right as waves crash against the shoreline with sand to the left. In the center above the horizon is a green circle.

The virtual reality technician set up a laptop, connected the virtual reality headset to the laptop, and turned on Steam VR and VIVE software needed to begin the scenario (Figure 1). The virtual reality technician assisted the participant to put on the headset and adjust the fit to their head. The headset fit like a ski mask covering the eyes with headphone‐type speakers near the ears. The virtual reality technician clicked on a calibration tool in the software that asked the participant to look at specific dots on the screen. The participant may have been prompted to adjust the headset based on the calibration test results. The virtual reality technician helped the participant to adjust the headset as needed. The technician then started the scenario in the language preferred by the participant. The participant saw information in text in English or Spanish, as they preferred, that informed them that they would be walking along a beach and should focus on the ball in front of them. The text on the screen informed the participant that, when they were ready, they should say “start” or look up until they see a ball (Figure 2). Once the scenario began, the participant saw, from a first‐person perspective, that they were strolling slowly along sand toward the shoreline of the ocean. The scenario lasted for 6‐min. There was a ball centered in front of them at the horizon. If they looked at the ball, it turned green. If they looked away, it turned red. The scenario included a 360‐degree point of view. If they chose, they could look around them behind them in both directions. To their right were crashing waves on the shoreline. To their far left was the Pacific Coast Highway with cars driving past. Seagulls flew past the participant and were near the shoreline throughout the scenario. They are moving slowly down the shoreline in the scenario. At the end of 6 min, the participant saw the screen go blank and then text showed them the percentage of time that they focused on the ball and how many times they looked away for immediate feedback.

### 2.3 Data Collection

Data included hospital‐level data on demographics: acute or long‐term injury, cause of injury, severity of injury, gender, age, insurance, language, and race/ethnicity). A baseline session as well as subsequent session screen recordings of Beach Stroll were used to measure eye fixation. The HTC‐VIVE Pro Eye headset software automatically calculates the percentage of the time that the participants’ eyes were focused on the ball (scores ranging from 1 (0%) to 5 (100%)).

The HTC Vive Pro Eye headset utilizes built‐in infrared pupil‐tracking technology with reported accuracy and precision for central gaze tasks (Schuetz and Fiehler 2022). Calibration was performed prior to each session using the manufacturer's standard 5‐ or 9‐point procedure, during which the participant was instructed to look at specific dots on the screen. The virtual reality technicians monitored calibration quality and adjusted the headset fit as needed. However, known limitations of the system include reduced spatial accuracy in peripheral vision, influence of head movement and individual facial configurations, potential interference from reflections or corrective lenses, and the need for careful calibration in dynamic environments (Sipatchin et al. 2021). These factors were considered and documented by the study team during each session.

All screen recordings were gathered by the virtual reality technicians, undergraduate health science students from California State University Channel Islands. Blood pressure and heart rate were measured immediately before and after each session using a blood pressure cuff and heart rate measurement shown on a device in the hospital room. If the device was not showing these measures, then they were taken manually by a registered nurse from the hospital who informed the virtual reality technician of the scores. Additionally, pre‐ and post‐surveys at each session were used to measure pain and anxiety. A 10‐point pain assessment tool that utilizes a numerical rating scale enhanced by functional word descriptors, color coding, and pictorial facial expressions matched to pain levels was completed pre and post each VR intervention. The Anxiety Verbal Rating Scale (VRS) 10‐point scale is a tool used to measure acute care anxiety using a verbal rating scale where 0 indicates no anxiety and 10 indicates the highest level of anxiety. The VRS‐10 was selected for this study because it is currently one of the assessment instruments routinely administered by physicians during clinical and hospital encounters. The VRS has a sensitivity of 89% and a specificity of 82%. It is moderately good at screening other common anxiety disorders—panic disorder (sensitivity 74%, specificity 81%) and post‐traumatic stress disorder (sensitivity 66%, specificity 81%). The VFAS assists patients in identifying their level of anxiety with a facial depiction of their anxiety level on a 10‐faces pictorial scale. All survey data were gathered by the virtual reality technician assisting the participant with the virtual reality equipment.

### 2.4 Analysis

Observation notes and participant quotes were analyzed using content analysis. Descriptive statistics were used for analysis of quantitative data.

## 3 Results: Patient Case Study

### 3.1 Patient Information

A white male in his mid‐30s had been hospitalized for over 30 days in the ICU and consented to participate within days of transfer to the step‐down ICU unit. He had sustained a severe TBI due to a motor vehicle accident near a beach in Southern California 60 days prior to study enrollment. He had been in a coma for 30 days following the injury and sustained a spinal cord injury resulting in paraplegia. At the time of admission, his Glasgow Coma Scale (GCS) score was 7, which is in the severe range consistent with the prolonged coma and subsequent neurological deficits. At baseline for the VR intervention, he demonstrated difficulty with visual fixation on day one and had to discontinue the session early on day two due to lower back phantom pain. He was concurrently receiving daily physical therapy to strengthen his physical recovery in preparation for discharge to an inpatient rehabilitation facility. The patient had Medi‐Cal insurance and was from a city approximately 45 min from the study hospital. While the patient's accident occurred at the beach, he still reported that the beach was his “happy place” and desired to complete the virtual reality scenario, which was beach‐themed. The patient had a large support system, including his mother, who was present almost every day in the hospital room, and friends who visited as well during the intervention. His mother was loving, attentive, and understandably anxious about her son's recovery.

### 3.2 Beach Stroll Frequency and Session Time With the Patient

The patient received a total of six sessions of the Beach Stroll intervention in English over the span of about a month prior to his hospital discharge to an inpatient rehabilitation from 3/19/24 to 4/22/24. There were two days that the patient was offered the intervention, but his caring mother, who was his primary caregiver and present each day of the intervention except one, did not want him to receive the intervention; she felt that it would be overstimulating. She turned off all the lights in his room those days and had classical music playing. On another day, the patient's mother did not want the patient to receive the intervention, but the patient expressed wanting to receive it. The unit charge nurse asked the patient's mother to clear the room to provide the patient with space to decide for himself since he was coherent and his own guardian. The patient participated in Beach Stroll that day.

**FIGURE 3:**
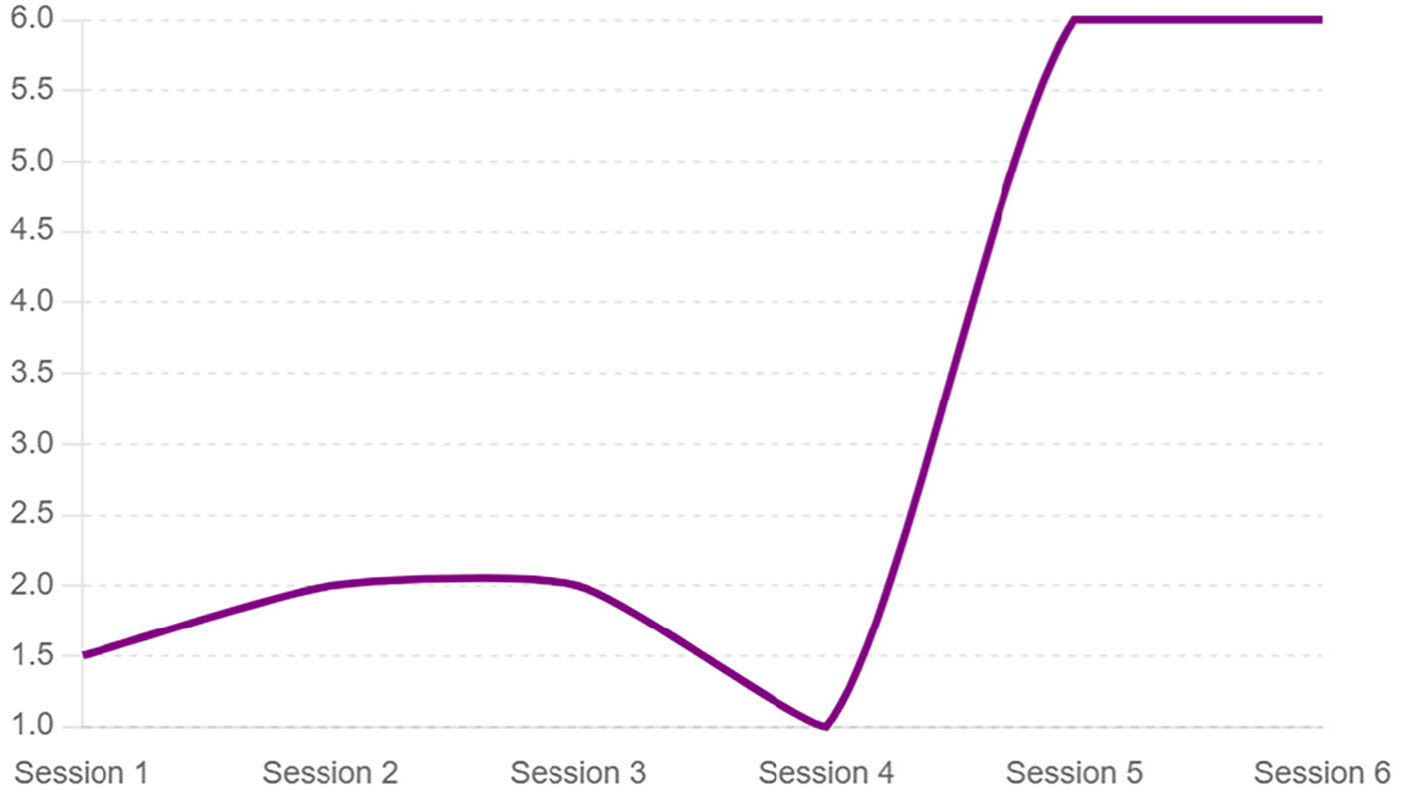
Patient Minutes in VR for Each Session. Alt text. The patient completed VR between 1 and 1.5 min in VR for the first 4 sessions. The patient increased to the full 6 min scenario for the fifth and sixth sessions.

While the intended session time was six minutes, the first few sessions ranged between 1–2 min each for the patient (Table 1; Figure 3). The patient was having back pain during the first and second sessions and kept wanting to change position. His physical therapist set him up in a reclining chair next to his hospital bed for those initial sessions. Future sessions were conducted in the hospital bed, and the patient did not complain of back pain nor stop the session early during those sessions.

**TABLE 1:** Minutes in VR and focus on object in each session.

Measure	Session 1	Session 2	Session 3	Session 4	Session 5	Session 6
Minutes in VR	1.5	2	2	1	6	6
Focus on object (fixation duration).	1	3	3	4	5	5

### 3.3 The Patient Results

**FIGURE 4:**
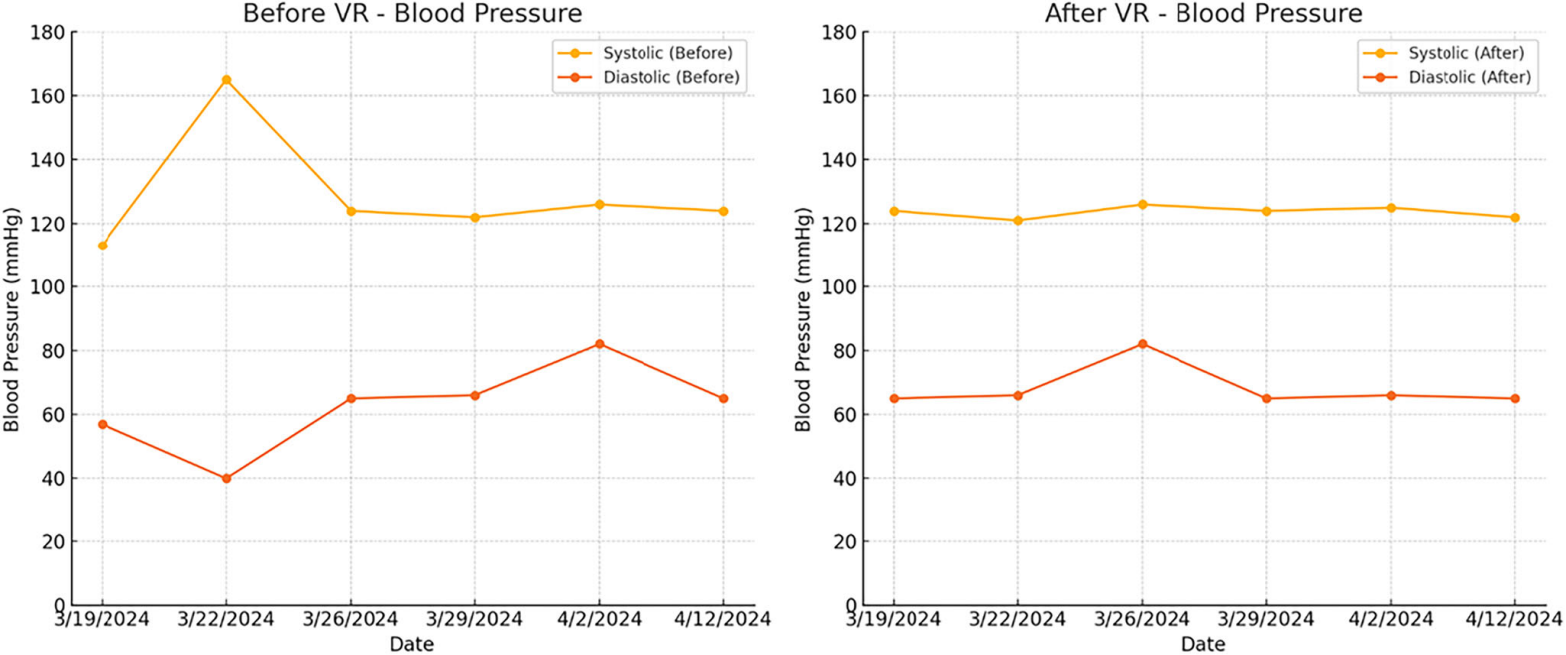
Patient's Blood Pressure. Alt text. There was not a consistent trend of the effects of VR on blood pressure. The patient's systolic blood pressure remained around 120 with an increase to 160 prior to the second session. After the second VR session, the patient's systolic blood pressure reduced to 120. After the third session, the patient's diastolic blood pressure went from a little over 60 to 80 after VR. After the fifth session, the patient's diastolic blood pressure went from 80 before VR to a little over 60 after VR.

**FIGURE 5:**
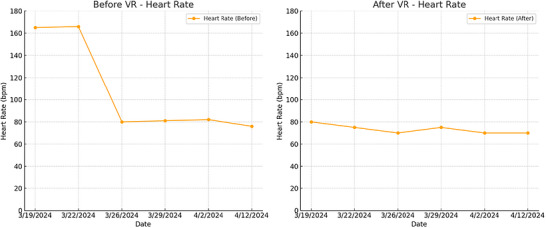
Patient's Heart Rate. Alt text. There was a consistent trend in a reduction in heart rate before and after VR for the patient. After the first and two sessions, there was a reduction of the patient's heart rate from about 160 to 80. The remainder of the sessions, the patient's heart rate went down slightly.

**FIGURE 6:**
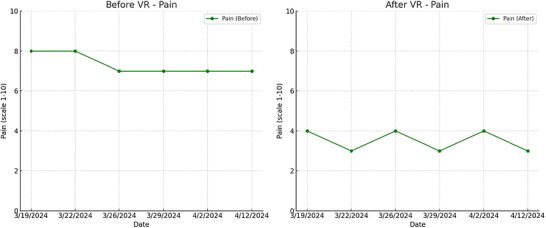
Patient's Pain. Alt text. The patient consistently reported a reduction in pain after each VR session.

**FIGURE 7:**
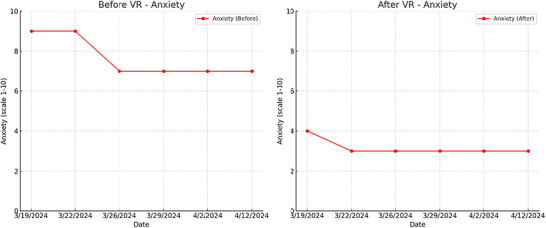
Patient's Anxiety. Alt text. The patient consistently reported a reduction in anxiety after each VR session.

**FIGURE 8:**
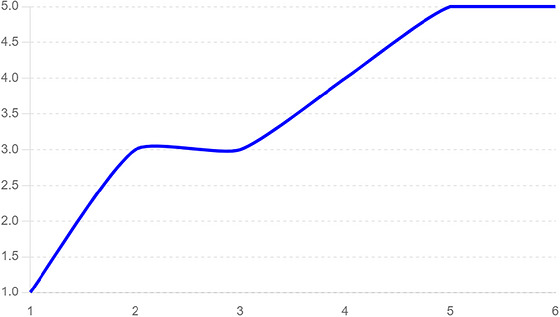
Patient's Ability to Focus. Alt text. The patient consistently increased his ability to focus in the VR scenario from the lowest amount to full focus by the fifth and sixth session.

Patient increased his ability to focus on the object in each consecutive session stabilizing at full focus during his last two sessions (Tables and Figures 3 and Figure 8). Fixation duration ranged from a score of 1(0%) at the baseline session to 5 (100%) at the fifth and final sessions. The patient's blood pressure (Figure 4) and heart rate (Figure 5) remained stable or decreased during each session (Table 2). Pain (Figure 6) and anxiety (Figure 7 ) decreased during each session as well (Table 2).

**TABLE 2:** Patient's pre‐ and post‐VR session blood pressure, heart rate, pain, and anxiety.

	Session 1	Session 2	Session 3	Session 4	Session 5	Session 6
Blood pressure (before)	112/58	162/40	123/62	120/63	124/80	123/63
Blood pressure (after)	123/63	120/63	123/80	122/63	122/63	121/63
Heart rate (before)	165	165	80	80	80	78
Heart rate (after)	80	78	70	78	70	70
Pain (before)	8	8	7	7	7	7
Pain (after)	4	3	4	3	4	3
Anxiety (before)	9	9	7	7	7	7
Anxiety (after)	4	3	3	3	3	3

### 3.4 Clinical Significance

Clinically, these physiological changes are meaningful. The reductions in heart rate (from peaks of 165 bpm to stable levels around 70–80 bpm) and stabilization or decreases in blood pressure suggest improved autonomic regulation and reduced sympathetic arousal during VR sessions. Pain scores decreased consistently from moderate‐severe levels (7–8) to mild levels (3–4), representing a clinically significant improvement that could facilitate greater participation in other therapies. Similarly, anxiety ratings dropped from high (7–9) to mild (3–4) levels, which is noteworthy in the acute post‐ICU setting where anxiety can impede engagement and recovery. These concurrent reductions in physiological stress markers and subjective distress support the potential of brief VR sessions to provide a calming effect while promoting visual engagement.

### 3.5 The Patient's Feedback

The patient's experience during six sessions of virtual reality (VR) for eye rehabilitation in the hospital was overwhelmingly positive. No adverse events were observed during the patient's six VR sessions. The patient tolerated the headset well, reported the experience as soothing and low‐stimulation, and did not experience cybersickness, dizziness, visual fatigue, or overstimulation.

The patient perceived the VR sessions as enjoyable and relaxing, verbally reporting a significant reduction in anxiety and increased comfort during the rehabilitation exercises. Patient described the experience as “the coolest thing I've done here [in the hospital],” with the sound being “really soothing” and the overall stimulation level being “low.” After the sixth session, regardless of her initial hesitance, the patient's mother expressed her gratitude, stating, “Thank you for your relaxing vibes.”

## 4 Discussion

These single‐case study findings align with the existing literature that highlights the potential of VR to enhance rehabilitation experiences. For instance, previous studies have shown that VR can improve engagement and motivation in rehabilitation exercises, provide real‐time feedback, and offer customized and immersive environments tailored to specific rehabilitation needs (Jiménez‐Rodríguez et al. 2023; Park et al. 2019). This single‐patient case report primarily demonstrates feasibility (successful bedside implementation in the acute post‐ICU setting with no major technical or safety issues) and acceptability (high patient enjoyment, soothing experience, and eventual family buy‐in). While positive trends were observed in visual fixation, physiological measures, pain, and anxiety, these findings should be interpreted as preliminary observations rather than evidence of clinical effectiveness. Larger, controlled studies are needed to establish efficacy and better isolate the contribution of VR from spontaneous recovery and concurrent therapies.

It is important to distinguish between VR applications in chronic versus acute TBI rehabilitation, as well as between general VR rehabilitation and VR specifically targeting visual dysfunction. Most prior VR research in TBI has focused on chronic phases (months to years post‐injury) and broader cognitive or motor outcomes rather than acute inpatient care (Aida et al. 2018; Watabe et al. 2019). Similarly, while several studies have examined VR for visual dysfunction, the majority were conducted in outpatient or laboratory settings with longer intervention protocols (Watabe et al. 2019; Linton et al. 2024). In contrast, the current case report demonstrates the feasibility of a brief, bedside VR eye‐tracking intervention in the acute post‐ICU hospital setting, addressing a notable gap in the literature where traditional rehabilitation options are often limited to physical and occupational therapy (Marklund et al. 2019). This acute application represents a novel extension of VR technology.

The importance of “buy‐in” from family and hospital staff was highlighted in the case study. The patient's mother's hesitance for his son to receive the intervention showed a lack of acceptance of the intervention from the patient's family; however, the charge nurse stepping in to ask for the mother to leave the patient's room to allow the patient to decide showed “buy‐in” from the hospital staff. The researchers of this study will continue to spend time presenting to and sharing information about the study, potential benefits of the virtual reality scenario, and updates on results of the study to ensure acceptance and support for the study.

This single‐case study highlights some limitations and areas for future research. While the patient experienced positive outcomes, the sample size is limited to a single individual, there is no control group, and there are no ophthalmological assessments. The patient also received physical therapy concurrently, and the intervention duration was relatively short. Immersive VR carries potential risks in neurologically vulnerable patients, including cybersickness, visual fatigue, dizziness, or overstimulation, particularly in the acute phase after TBI. Future studies should systematically monitor for these adverse events using standardized tools and ensure appropriate patient screening (e.g., excluding those with severe motion sensitivity or uncontrolled seizures). Future research should also involve larger, more diverse samples and longer intervention periods to better understand the long‐term efficacy and generalizability of VR for eye rehabilitation in TBI patients. Additionally, standardized protocols for VR rehabilitation need to be developed to ensure consistency and reliability in treatment approaches and outcomes (Raphanel et al. 2018).

It is important to note that because spontaneous neurological recovery is expected during the early post‐ICU period, the improvements observed in visual fixation, physiological stability, pain, and anxiety cannot be attributed solely to the VR intervention. Natural recovery processes, concurrent physical therapy, and other standard care likely contributed to the patient's progress. This case report therefore demonstrates feasibility and promising preliminary observations rather than definitive causal efficacy. Future studies incorporating control conditions, repeated baseline assessments, or randomized designs will be essential to better isolate the specific contribution of VR‐based eye‐tracking rehabilitation.

It is also important to consider that reductions in anxiety observed during and after VR sessions may have independently contributed to improvements in attention and visual fixation performance. Anxiety is known to impair cognitive and oculomotor function in TBI patients (Delmonico et al. 2022). Therefore, the calming effect of the immersive, low‐stimulation Beach Stroll scenario may have indirectly supported better eye‐tracking performance by reducing anxiety‐related interference, rather than solely through direct visual training effects. This potential confounding factor underscores the value of future studies that measure and control for anxiety levels more systematically.

The practical implications of these preliminary findings suggest that VR may warrant further exploration as a potential adjunct in rehabilitation programs for TBI patients to support visual functions while also addressing emotional well‐being. The customization of VR scenarios, such as the Beach Stroll used in this study, can cater to individual patient preferences and needs, potentially making the rehabilitation process more enjoyable. Furthermore, VR's potential for home‐based rehabilitation could reduce the need for frequent clinic visits, making it a convenient option worthy of future investigation (Jiménez‐Rodríguez et al. 2023).

The feasibility of implementing this VR intervention across different hospitals appears promising but would require attention to several practical barriers. Equipment cost (VR headset) remains a significant initial investment, although prices are expected to decrease with advancing technology. Personnel training for virtual reality technicians (undergraduate students in this study) would be necessary, along with supervision by clinical staff. Workflow integration into busy acute trauma units and infection‐control considerations (e.g., electronically safe headset cleaning protocols between patients) would also need to be addressed. Coordination with physical therapy, nurses, and other staff is key to obtaining and sustaining “buy‐in” from hospital clinical staff. Future multi‐site studies should evaluate these implementation factors to facilitate broader adoption in diverse hospital settings.

In conclusion, this single‐patient case report provides preliminary support for the feasibility and acceptability of brief VR eye rehabilitation in the acute post‐ICU TBI setting. Larger, controlled studies are needed to establish efficacy and better isolate the contribution of VR from spontaneous recovery and concurrent therapies. Future research would be strengthened by a prospective randomized controlled design comparing VR‐assisted eye‐tracking rehabilitation with conventional visual rehabilitation or standard care in acute TBI patients. Such trials could better establish efficacy, optimal dosing, and generalizability across diverse patient populations and hospital settings. In particular, studies should incorporate standardized neuropsychological, functional, and quality‐of‐life assessments and longer‐term follow‐up to evaluate whether improvements extend beyond eye fixation to broader cognitive and daily functioning domains. Inclusion of before‐and‐after ophthalmological or neuro‐ophthalmological assessments would also considerably strengthen future work.

## Author Contributions

**Javier Romero**: supervision, methodology. **Savanna Monson**: data curation, writing – original draft. **Graal Diaz**: writing – original draft, methodology, data curation, supervision. **Nathan Oh**: methodology, supervision. **Jaylyn Aragon**: writing – original draft, data curation. **Kristen Linton**: conceptualization, investigation, writing – original draft, writing – review and editing, methodology, formal analysis, software, project administration, supervision, data curation. **Bahareh Abbasi**: software, resources.

## Funding

The authors would like to thank California State University Channel Islands and the California Department of Rehabilitation for funding this project.

## Ethics Statement

This study has been approved by the California State University Channel Islands and Ventura County Medical Center Institutional Review Boards.

## Conflicts of Interest

The authors declare no conflicts of interest.

## Data Availability

Data is available at the request of the corresponding author.
